# Kaposi’s Sarcoma-Associated Herpesvirus ORF7 Is Essential for Virus Production

**DOI:** 10.3390/microorganisms9061169

**Published:** 2021-05-28

**Authors:** Yuki Iwaisako, Tadashi Watanabe, Mizuki Hanajiri, Yuichi Sekine, Masahiro Fujimuro

**Affiliations:** Department of Cell Biology, Kyoto Pharmaceutical University, Kyoto 607-8412, Japan; kd21002@ms.kyoto-phu.ac.jp (Y.I.); twatanab@med.u-ryukyu.ac.jp (T.W.); h.m1zu@icloud.com (M.H.); sekine@mb.kyoto-phu.ac.jp (Y.S.)

**Keywords:** kaposi’s sarcoma-associated herpesvirus, terminase, lytic replication, capsid formation, ORF7, herpesvirus

## Abstract

Kaposi’s sarcoma-associated herpesvirus (KSHV) causes Kaposi’s sarcoma, primary effusion lymphoma (PEL), and multicentric Castleman disease. Although capsid formation and maturation in the alpha-herpesvirus herpes simplex virus 1 are well understood, these processes in KSHV remain unknown. The KSHV ORF7, encoding the viral terminase (DNA cleavage and packaging protein), is thought to contribute to capsid formation; however, functional information is lacking. Here, we investigated the role of ORF7 during KSHV lytic replication by generating two types of ORF7 knock-out (KO) mutants (frameshift-induced and stop codon-induced ORF7 deficiency), KSHV BAC16, and its revertants. The results revealed that both ORF7-KO KSHVs showed significantly reduced viral production but there was no effect on lytic gene expression and viral genome replication. Complementation assays showed virus production from cells harboring ORF7-KO KSHV could be recovered by ORF7 overexpression. Additionally, exogenously expressed ORF7 partially induced nuclear relocalization of the other terminase components, ORF29 and ORF67.5. ORF7 interacted with both ORF29 and ORF67.5, whereas ORF29 and ORF67.5 failed to interact with each other, suggesting that ORF7 functions as a hub molecule in the KSHV terminase complex for interactions between ORF29 and ORF67.5. These findings indicate that ORF7 plays a key role in viral replication, as a component of terminase.

## 1. Introduction

Kaposi’s sarcoma-associated herpesvirus (KSHV), also known as human herpesvirus 8 (HHV-8), is classified as a rhadinovirus in the *gamma-herpesvirinae* subfamily [1,2]. KSHV was first discovered in 1994 in a Kaposi’s sarcoma lesion from a patient with AIDS [1]. It was subsequently found to be closely associated with Kaposi’s sarcoma, primary effusion lymphoma (PEL), multicentric Castleman’s disease, and KSHV-associated inflammatory cytokine syndrome [2,3,4,5]. KSHV establishes a life-long infection in human B-cells or vascular endothelial cells and exists in either a latent or lytic state. During latent infection, the KSHV genome circularizes to form an episome in the nucleus and expresses several latent-associated gene products, which contribute to the promotion of cell proliferation and anti-apoptosis activity [6]. KSHV shifts its life cycle from a latent to a lytic infection by the expression of a replication and transcription activator (RTA/ORF50). During the lytic cycle, most lytic-related genes are expressed in the infected cell, and virions are assembled and then egress from the infected cell. As with other herpesviruses, KSHV virions consist of a linear, double-stranded DNA genome enclosed within an icosahedral capsid shell, tegument proteins, and a lipid bilayer envelope spiked with glycoproteins [7].

The fundamental structure of virus particles and the process of viral capsid formation are thought to be analogous among the human herpesviruses [8]. The process of KSHV encapsidation in an infected cell nucleus remains largely unknown, whereas in herpes simplex virus 1 (HSV-1) it is well understood [9,10]. KSHV genes correspond to homologs in HSV-1; their common names are listed in Table 1. In the case of HSV-1, the first step in capsid formation is the assembly in the nucleus of the procapsid, which consists of an external and an internal capsid shell [9,10]. A major capsid protein and an assembly protein form a heterodimer; these heterodimers then self-assemble to form a procapsid. The external capsid proteins (containing a large amount of major capsid proteins, small capsid proteins, triplex proteins, portal capping proteins, and more) form the spherical shell (i.e., the external capsid shell) of a procapsid. The internal capsid proteins (assembly proteins and considerably lower amounts of previral protease) form the spherical internal backbone (i.e., the internal capsid shell) of a procapsid. The next step in encapsidation is the viral protease-mediated processing of internal scaffold proteins of the procapsid. The viral protease cleaves the C-terminal region of assembly proteins and eliminates the internal capsid proteins (assembly proteins) from the interior cavity of the procapsid [11]. Thereafter, a spherical external capsid shell causes a conformational change in the capsid’s morphology, and it becomes an icosahedral capsid [12,13].

The last step in encapsidation in the nucleus is the cleavage of viral DNA genomes and insertion of appropriate genome units into the capsid cavity. HSV-1 genomes are replicated as concatemeric intermediate DNA that forms by connecting multiple genomes. The mechanism underlying this remains largely unknown; however, the terminases (also known as DNA cleavage and packaging proteins) are known to be necessary for the cleavage at the terminal repeats of intermediate viral DNA concatemers and the insertion of appropriate unit-lengths of genome into the capsid [14,15,16,17,18,19]. HSV-1 DNA cleavage and packaging into the procapsid is mediated by a terminase complex (consisting of UL15, UL28, and UL33 proteins) [20,21,22], portal protein (UL6), and capsid-associated tegument complexes (consisting of UL25, UL17, and UL36) [10,23]. A UL28 deletion mutant of HSV-1 was unable to cleave the concatemeric viral genomes [24]. The cleaved HSV-1 genome is inserted into the capsid cavity of the procapsid via the portal protein [10,23]. The completed capsid (i.e., nucleocapsid), with its viral DNA and mature external capsid, is exported out of the nucleus through the nuclear membrane, obtaining tegument proteins and a lipid bilayer envelope. It can be seen, then, that the factors involved in capsid assembly and DNA packaging in HSV-1 have been intensively investigated. However, these factors in KSHV remain unknown. Therefore, in this study, we investigated KSHV ORF7, a homolog of HSV-1 UL28. We established ORF7-deficient KSHV and evaluated the physiological role of KSHV ORF7 during viral replication.

## 2. Materials and Methods

### 2.1. Plasmids

The coding DNA fragments of KSHV ORF7, ORF17 and ORF67.5 were amplified from BAC16 DNA (accession number: GQ994935) by PCR using KOD high-fidelity DNA polymerase (Toyobo, Osaka, Japan), and the coding DNA of ORF29 was amplified from cDNA from lytic-induced iSLK-WT KSHV BAC16 cells. All used primers for construction of expression plasmids are shown in Table 2. Each DNA fragment was cloned into pCI-neo-2xS, pCI-neo-3xFLAG, pCI-neo-5xHA, or pCI-neo-6xmyc vectors, which had been generated by inserting oligonucleotides encoding two repeats of an S-tag peptide, three repeats of a FLAG-tag, five repeats of an HA-tag, or six repeats of a myc-tag sequence, respectively, into the pCI-neo mammalian expression vector (Promega, WI, USA).

### 2.2. Construction of ORF7-KO KSHV BAC16

Wild-type (WT) KSHV BAC16 (WT-BAC16) was kindly provided by Jae U. Jung. ORF7-KO recombinant KSHV BAC16 (frameshift-induced ORF7-BAC16 (FS-ΔORF7-BAC16) and stop codon-induced ORF7 (ST-ΔORF7-BAC16)) were generated from WT-BAC16 using a two-step markerless Red recombination system [25,26,27]. Its revertants, ST Rev-BAC16 and FS Rev-BAC16, were generated from ST-ΔORF7-BAC16 and FS-ΔORF7-BAC16, respectively. This mutagenesis of BAC clones was performed according to previously described protocols [25], using the mutagenesis primers shown in Table 2. Insertions and deletions of kanamycin-resistance cassettes (KanR) in each mutant were analyzed by digestion of BglII and agarose gel electrophoresis. ORF7 and mutated sites in each BAC clone (ORF7 coding region (nt6609–nt8696; accession number: GQ994935)) were confirmed by Sanger sequencing.

### 2.3. Establishment of Doxycycline-Inducible Recombinant KSHV-Expressing Cells

To obtain efficient recombinant KSHV-producing cells, tetracycline/doxycycline (Dox)-inducible (Tet-on) RTA/ORF50-expressing SLK cells (iSLK) were used as virus-producing cells [28]. For maintenance, iSLK cells were cultured in growth medium containing 1 μg/mL of puromycin (InvivoGen, CA, USA) and 0.25 mg/mL of G418 (Nacalai Tesque, Kyoto, Japan). WT-BAC16 (10 μg) or its mutants (ΔORF7 FS-BAC16, ΔORF7 ST-BAC16, FS Rev-BAC16, and ST Rev-BAC16) were transfected into iSLK cells. The iSLK cells (1 × 10^6^ cells) were transfected using the calcium phosphate method. The transfected cells were selected under 1000 μg/mL of hygromycin B (Wako, Osaka, Japan) to establish doxycycline-inducible recombinant KSHV-producing cell lines (iSLK-WT, iSLK-FS-ΔORF7, iSLK-ST-ΔORF7, iSLK-FS Rev, and iSLK-ST Rev cells).

### 2.4. Measurement of Virus Production and Viral DNA Replication

For quantification of virus production, KSHV virions in culture supernatant were quantified as previously described [28,29]. Briefly, iSLK-WT, iSLK-FS-ΔORF7, iSLK-ST-ΔORF7, iSLK-FS Rev, or iSLK-ST Rev cells were treated with 8 μg/mL of doxycycline (Dox) and 1.5 mM of sodium butyrate (NaB) for 72 h to induce to lytic replication, and then the culture supernatants including recombinant KSHV were harvested. The culture supernatants (220 μL) were treated with DNase I (New England Biolabs, MA, USA) to obtain only enveloped and encapsidated viral genomes. Viral DNA was purified and extracted from 200 μL of the DNase I-treated culture supernatant using a QIAamp DNA Blood Mini Kit (Qiagen, CA, USA). To quantify the viral DNA copies, SYBR green real-time PCR was performed using KSHV-encoded ORF11-specific primers, noted in Table 2. For measurement of KSHV genome replication, each KSHV-producing cell line was treated with Dox and NaB for 72 h to induce lytic replication then harvested. Total cellular DNA containing the KSHV genomes was purified and extracted from washed cells using the QIAamp DNA Blood Mini Kit (Qiagen). The number of cellular KSHV genome copies was determined using SYBR green real-time PCR and normalized to the total number of DNA copies.

### 2.5. RT Real-Time PCR (RT-qPCR)

iSLK cells were treated with 8 μg/mL of doxycycline (Dox) and 1.5 mM of sodium butyrate (NaB) for 72 h to induce lytic replication. Total RNA was purified and extracted from cells using RNAiso Plus (TAKARA, Osaka, Japan). cDNA was synthesized by ReverTra Ace qPCR RT kit (TOYOBO, Osaka, Japan) and subjected to SYBR green real-time PCR. The sequences of RT-qPCR primer sets were noted in Table 2. Relative mRNA expression levels were determined by GAPDH expression and ΔΔCt methods.

### 2.6. Complementation Assay

The iSLK cells (1 × 10^5^ cells) were transfected with either pCI-neo-3xFLAG empty plasmid or 3xFLAG-tagged ORF7 plasmid using Screenfect A plus (Wako, Tokyo, JAPAN), according to the manufacturer’s instructions, then simultaneously cultured with medium containing 8 μg/mL of Dox and 1.5 mM of NaB to induce lytic replication. After 3 days, culture supernatant was harvested and KSHV genomes were evaluated using real-time PCR. In addition to measuring KSHV genome, the virus titers in culture supernatants were also evaluated. The iSLK cells (1 × 10^6^ cells) were transfected with ORF7-3xFLAG plasmid using Screenfect A plus (Wako) and treated with Dox and NaB. After 72 h, culture supernatants were collected and centrifuged at 15,000 rpm 10 min at room temperature, then each supernatant (200 μL) was mixed with 1 × 10^4^ trypsinized HEK293T or HeLa cells. Polybrene (Sigma-Aldrich, MO, USA) was added to the cell mixture (8 μg/mL final concentration), and cells were placed into a 96-well plate. The 96-well plate was centrifuged at 1200× *g* for 1 h at room temperature. After 24 h, GFP-positive cells were counted by fluorescence microscopy.

### 2.7. Antibodies

Anti-FLAG mouse mAb (M185), anti-HA tag mouse mAb (M180), anti-S tag rabbit pAb (PM021) (MBL, Nagoya, Japan), and anti-beta-actin mouse mAb (sc-69879) (Santa Cruz Biotechnology, Dallas, TX, USA) were used as the primary antibodies. Anti-mouse IgG-HRP (NA931) (GE Healthcare, Buckinghamshire, UK) and anti-rabbit IgG-HRP (#7074) (Cell Signaling Technology, Danvers, MA, USA) were used as the secondary antibody for the Western blotting. Anti-S tag mouse mAb (71549) (Merck, Darmstadt, Germany), anti-HSP90-beta goat pAb (sc-1057), anti-S tag rabbit pAb (sc-802), anti-myc tag mouse mAb (sc-40) (Santa Cruz Biotechnology), and anti-FLAG mouse mAb conjugated with Alexa Fluor 488 (M185-A48) (MBL) were used as the primary antibodies. The secondary antibodies conjugated to Alexa Fluor 488 (A11034), 594 (A21203), and 647 (A21447) were obtained from Invitrogen (Waltham, CA, USA) for the IFA.

### 2.8. Western Blotting and Immunofluorescence Assay (IFA)

Western blot analyses were performed as previously described [29]. For the IFA, HeLa cells (1 × 10^5^) on a glass slide were transfected with 4 μg plasmid DNA with 12 μg polyethylenimine hydrochloride/PEI MAX (Polysciences, Inc., Warrington, PA, USA); the medium was replaced with fresh medium after 3 h. Cells were cultured for a further 15 h then fixed with 4% paraformaldehyde at room temperature for 15 min and permeabilized with 0.1% Triton X-100 in PBS. The samples were treated with 10% fetal bovine serum in PBS and incubated with primary antibodies at 37 °C for 1 h. After washing with PBS-T (0.1% Tween 20 in PBS), cells were incubated with secondary antibodies at 37 °C for 1 h. After washing six times with PBS-T, cells were further stained with anti-FLAG mouse mAb conjugated with Alexa Fluor 488 at 37 °C for 1 h. Stained samples were embedded in Fluoro-KEEPER Antifade Reagent, Non-Hardening Type with DAPI (12745-74) (Nacalai Tesque, Kyoto, Japan) and observed under a confocal LSM 800 microscope (Carl Zeiss, Oberkochen, Germany) using the LSM software (Carl Zeiss).

### 2.9. Pulldown Assays

HEK293T cells (2 × 10^6^) were transfected with 12 μg plasmid DNA and 120 μg polyethylenimine hydrochloride/PEI MAX (Polysciences) and subsequently cultured for 18 h. Cells were lysed with 1.5 mL lysis buffer (50 mM Tris/HCl pH 8.0, 120 mM NaCl, 1% glycerin, 0.2% Nonidet P-40, and 1 mM dithiothreitol). Cell extracts were incubated with 20 µL S-protein-immobilized agarose beads for 1 h, and then the beads were washed three times with lysis buffer. Clarified beads were resuspended in 30 µL SDS-PAGE sample buffer with 2% 2-ME. Precipitates were probed with anti-FLAG, anti-HA, or anti-S tag antibody after Western blotting.

### 2.10. Statistics

A two-tailed Student’s t-test was used to explore differences between the groups. *p*-values are presented in the figures.

## 3. Results

### 3.1. Construction of Two Types of ORF7-KO KSHV BACs and Their Revertants

**Figure 1:**
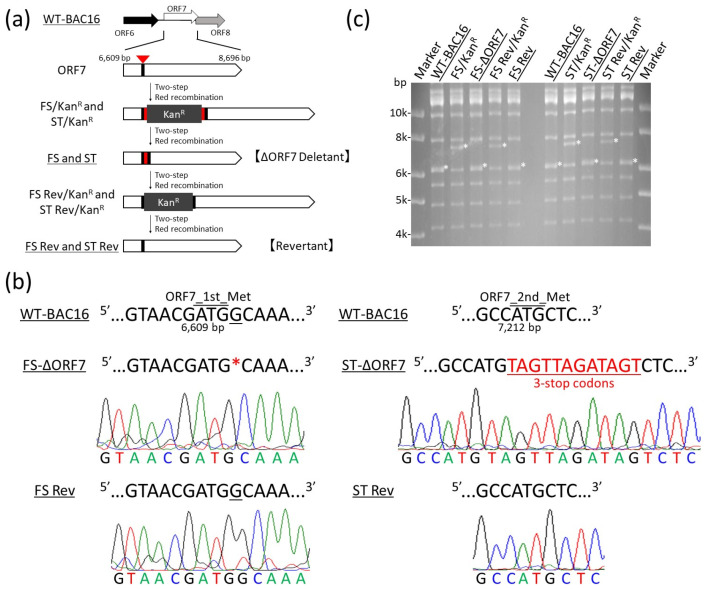
Construction of the frameshift-induced ORF7 and the stop codon-induced ORF7 KSHV BAC16 and their revertants. (**a**) Schematic illustration of the KSHV genome, including the ORF7 coding region. Two different types of ORF7-KO mutations (i.e., a frameshift using a one-base deletion and an insertion of three stop codons) were generated in the ORF7 coding region (nt6609–nt8696; accession number: GQ994935) of wild-type (WT)-KSHV BAC16 clones. The frameshift and insertion mutants were designated FS-ΔORF7-BAC16 and ST-ΔORF7-BAC16, respectively. Additionally, revertant clones, FS Rev-BAC16 and ST Rev-BAC16, were generated from FS-ΔORF7-BAC16 and ST-ΔORF7-BAC16, respectively, by replacement of the mutation site with the original DNA sequence. (**b**) The neighboring DNA sequence of the mutation sites in FS-ΔORF7-BAC16, ST-ΔORF7-BAC16, and their revertant BAC16 clones. (**c**) An image of the gel from agarose gel electrophoresis of the recombinant KSHV BAC16 digested with BglII enzyme. The asterisks indicate insertion or deletion of a kanamycin-resistance cassette in each BAC clone. Original images of the blotting are shown in Supplementary Figure S1.

To elucidate the biological role played by the ORF7 of KSHV, we constructed two types of ORF7-deficient mutants, i.e., frameshift-induced ORF7-BAC16 (designated FS-ΔORF7-BAC16) and stop codon-induced ORF7-BAC16 (designated ST-ΔORF7-BAC16) from wild-type (WT) KSHV BAC16 (WT-BAC16) using a two-step markerless Red recombination method (Figure 1a) [26,27]. For FS-ΔORF7-BAC16, we deleted the first base “G” of the second codon (GCA) after the first codon (ATG) in the ORF7 coding region of WT-BAC16 (Figure 1b). Therefore, a frameshift of one base occurred at the ORF7 translation in FS-ΔORF7-BAC16, and then a 45 bp (coding 14 amino acids) random sequence RNA was transcribed. We consider the effect of this frameshift mutation on the polyadenylation site of ORF6 gene to be unknown because the polyadenylation site of the ORF6 gene is located 381 b downstream of the frameshift mutation site, the second codon (GCA) [30]. Therefore, we generated an additional mutant, the stop codon-induced ORF7-BAC16 (designated ST-ΔORF7-BAC16), in which three stop codons were inserted into the second Met (ATG) of the ORF7 coding region (2088 b). The ORF7 N-terminal region (202 amino acids) might be translated from ST-ΔORF7-BAC16. We also created revertants, which removed each added mutation. FS Rev-BAC16 and ST Rev-BAC16 were generated from FS-ΔORF7-BAC16 and ST-ΔORF7-BAC16, respectively. The insertion and deletion of the kanamycin-resistance cassette (KanR) was confirmed by restriction enzyme (BglII) digestion and agarose gel electrophoresis (Figure 1c). The sequences of each added mutation and each revertant were confirmed by Sanger sequencing (Figure 1b).

### 3.2. ORF7 Is Essential for Virus Production but Not Lytic Gene Expression and DNA Replication in KSHV

To efficiently induce recombinant KSHV, tetracycline/doxycycline (Dox)-inducible (Tet-on) RTA/ORF50-expressing SLK cells (iSLK) were used as virus-producer cells [28]. When iSLK cells are treated with Dox and NaB, RTA expression is induced in iSLK cells; i.e., lytic replication and subsequently virus production are induced in the case of BAC16-transfected iSLK cells. WT-BAC16, FS-ΔORF7-BAC16, ST-ΔORF7-BAC16, FS Rev-BAC16, and ST Rev-BAC16 clones were transfected into the iSLK cell line, and stable cells harboring BAC16 were selected by hygromycin B. Then, Dox-inducible recombinant KSHV-expressing polyclonal cell lines were established: iSLK-WT, iSLK-FS-ΔORF7, iSLK-ST-ΔORF7, iSLK-FS Rev, and iSLK-ST Rev cells.

**Figure 2:**
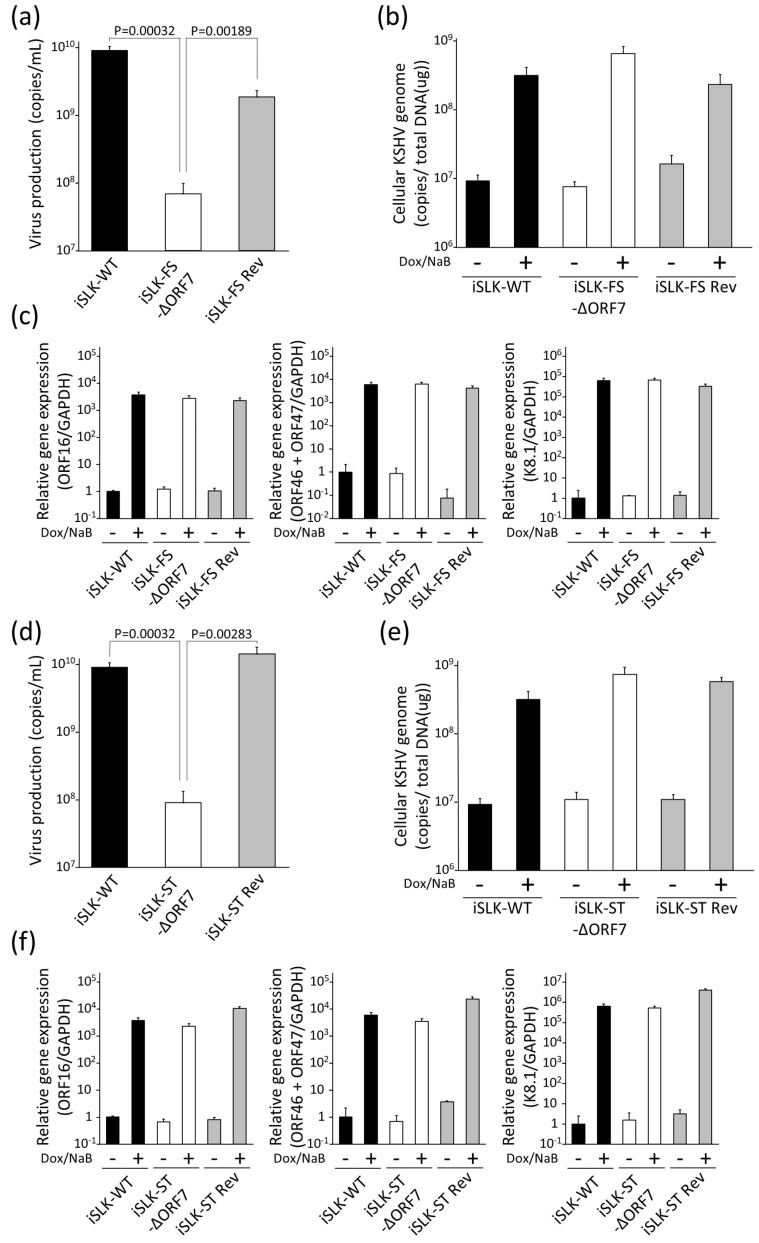
The effects of ORF7 deficiency on KSHV lytic replication. (**a**–**c**) Extracellular virus production and intracellular viral DNA replication and lytic gene expression in iSLK cells harboring WT-KSHV-BAC16, frameshift-induced ORF7-KSHV-BAC16, and revertant KSHV-BAC16. The iSLK-WT, iSLK-FS ΔORF7, and iSLK-FS Rev cell lines were cultured for 72 hours with medium containing Dox and NaB to induce lytic replication. (**a**) KSHV genome copies of packaged viral particles in culture supernatants were measured using real-time PCR. (**b**) The quantities of intracellular viral DNA of Dox- and NaB-treated iSLK cells were measured using real-time PCR and normalized by the total amount of DNA. (**c**) The quantities of mRNA expression levels of viral lytic genes: IE, ORF16 (vBcl-2); E, ORF46 (uracil DNA glycosylase) and ORF47 (glycoprotein L); L, K8.1 (glycoprotein) in lytic-induced iSLK-WT, iSLK-FS ΔORF7, and iSLK-FS Rev cell lines. Each iSLK cell line was treated with or without 8 μg/mL Dox and 1.5 mM NaB, and cells were harvested at 72 h post-treatment. Total RNA purified from cells was subjected to RT real-time PCR. Evaluated lytic genes were normalized by GAPDH mRNA. The values obtained from Dox- and NaB-untreated iSLK-WT cells were defined as 1.0. (**d**–**f**) Extracellular virus production and intracellular viral DNA replication in iSLK cells harboring WT-KSHV-BAC16, stop codon-induced ORF7-KSHV-BAC16, and revertant KSHV-BAC16. The iSLK-WT, iSLK-ST ΔORF7, and iSLK-ST Rev cell lines were cultured with Dox and NaB for 72 h. (**d**) KSHV genome copies of viral particles in culture supernatants were measured using real-time PCR. (**e**) The quantities of intracellular viral DNA of Dox- and NaB-treated iSLK cells were normalized by the total amount of DNA. (**f**) The quantities of mRNA expression levels of viral lytic genes of Dox- and NaB-treated iSLK cells were measured using RT real-time PCR and normalized by GAPDH mRNA. The values obtained from Dox- and NaB-untreated iSLK-WT cells were defined as 1.0.

To evaluate whether ORF7 is important for KSHV replication, virus production in iSLK-ORF7-deficient KSHV cells was analyzed first. iSLK-WT, iSLK-FS-ΔORF7, iSLK-ST-ΔORF7, iSLK-FS Rev, and iSLK-ST Rev cells were treated with Dox and NaB to induce lytic replication, and the culture supernatants were harvested. The amount of recombinant KSHV in cell culture supernatants was measured using real-time PCR (Figure 2a,c). The quantity of virus produced by iSLK-FS-ΔORF7 and iSLK-ST-ΔORF7 cells was approximately 100-fold lower than the quantity produced by iSLK-WT, iSLK-FS Rev, and iSLK-ST Rev cells. Next, the replication of intracellular virus genome (cell-associated virus genome) and lytic gene expression in iSLK-FS-ΔORF7 and iSLK-ST-ΔORF7 cells were analyzed. iSLK-WT, iSLK-FS-ΔORF7, iSLK-ST-ΔORF7, iSLK-FS Rev, and iSLK-ST Rev cells were treated with Dox and NaB, and lytic-induced cells were harvested. The quantities of viral genome within WT KSHV, ΔORF7-KSHV, and Rev KSHV in iSLK cells were measured using real-time PCR (Figure 2b,d). In contrast to virus production, there was no significant difference in intracellular KSHV DNA levels among WT-KSHV, ORF7-deficient KSHV, and Rev KSHV-producing cells. We also elucidated the effects of the ORF7 defect on the expression of lytic genes. The mRNA expression of immediate early (IE) (ORF16), early (E) (ORF46 and ORF47), and late (L) (K8.1) genes was evaluated by RT real-time PCR in lytic-induced iSLK-WT, iSLK-FS-ΔORF7, iSLK-FS Rev, iSLK-ST-ΔORF7, and iSLK-ST Rev cells. Expression levels of IE, E, and L genes were comparable among iSLK-WT, iSLK-ΔORF7, and iSLK-Rev cells. Moreover, we confirmed that ORF7 expression did not influence the lytic replication of KSHV (data is shown in Supplementary Figure S2). These data indicate that ORF7 plays an important role in virus production but not intracellular DNA replication in KSHV.

### 3.3. Rescue of Virus Production in iSLK-ΔORF7 Cells by ORF7 Overexpression

**Figure 3:**
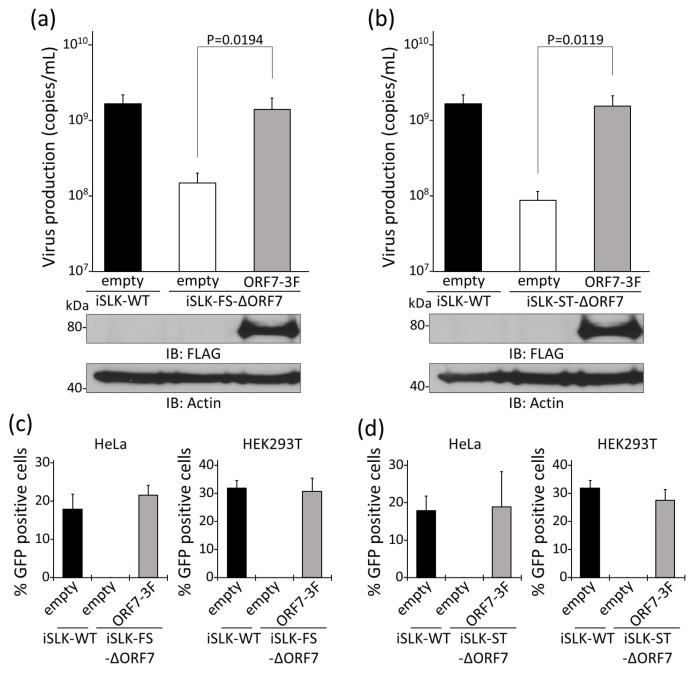
Virus production in iSLK cells harboring ORF7-deficient KSHV was recovered by exogenous ORF7 expression. Rescue of virus production in iSLK-FS ΔORF7 cells (**a**) and iSLK-ST ΔORF7 cells (**b**) by exogenous ORF7 expression. iSLK-FS ΔORF7 or iSLK-ST ΔORF7 cells were transiently transfected with ORF7-3xFLAG plasmid (or empty plasmid) and simultaneously cultured with medium containing Dox and NaB for 72 h. At 72 h after treatment and transfection, levels of KSHV genome copies of KSHV virions in culture supernatants were determined using real-time PCR. Exogenous ORF7 expression in ORF7-3xFLAG-transfected and Dox-induced iSLK cells was confirmed by Western blotting using anti-FLAG antibody. Original images of the Western blotting are shown in Supplementary Figure S2. (**c**,**d**) Evaluation of infectious virion in culture supernatants of ORF7-3xFLAG-transfected iSLK-FS ΔORF7 cells (**c**) and iSLK-ST ΔORF7 cells (**d**). At 72 h after Dox treatment and transfection, collected culture supernatants were inoculated with HeLa and HEK293T cells for infection. At 72 h post-infection, GFP-positive cells were counted by fluorescence microscopy. (**a**–**d**) “empty” and “ORF7-3F” mean the empty plasmid and ORF7-3xFLAG plasmid, respectively.

To obtain further evidence that the reduction in virus production in iSLK-FS-ΔORF7 and iSLK-ST-ΔORF7 cells was caused by ORF7 deficiency, we tested whether exogenous ORF7 expression could rescue virus production in iSLK-FS-ΔORF7 and iSLK-ST-ΔORF7 cells. iSLK-FS-ΔORF7 and iSLK-ST-ΔORF7 cells were transfected with either empty plasmid or ORF7-3xFLAG expression plasmid. Transfected cells were treated with Dox and NaB. Subsequently, culture supernatants were subjected to real-time PCR to measure virus production. Virus production in iSLK-FS-ΔORF7 and iSLK-ST-ΔORF7 cells recovered significantly when ORF7-3xFLAG plasmid was exogenously overexpressed (Figure 3a,b). The protein expression of 3xFLAG-tagged ORF7 was confirmed by Western blotting using anti-FLAG antibody (the lower panels of Figure 3a,b). In addition to measuring viral DNA, we also evaluated the virus titers in culture supernatants. The culture medium was added to HeLa and HEK293T cells, and the numbers of GFP-positive cells were counted to evaluate the infectious virion production. As a result, the infectivities of produced viruses from iSLK-FS-ΔORF7 (Figure 3c) and iSLK-ST-ΔORF7 cells (Figure 3d) were recovered by exogenous ORF7-3xFLAG overexpression. These results provide further evidence to the data shown in Figure 2 and support a model that ORF7 is crucial for KSHV virus production.

### 3.4. ORF29 and ORF67.5 Are Partially Relocalized into Nuclei from the Cytosol by ORF7 and ORF7 Colocalizes with ORF29 and ORF67.5 in the Nuclei

**Figure 4:**
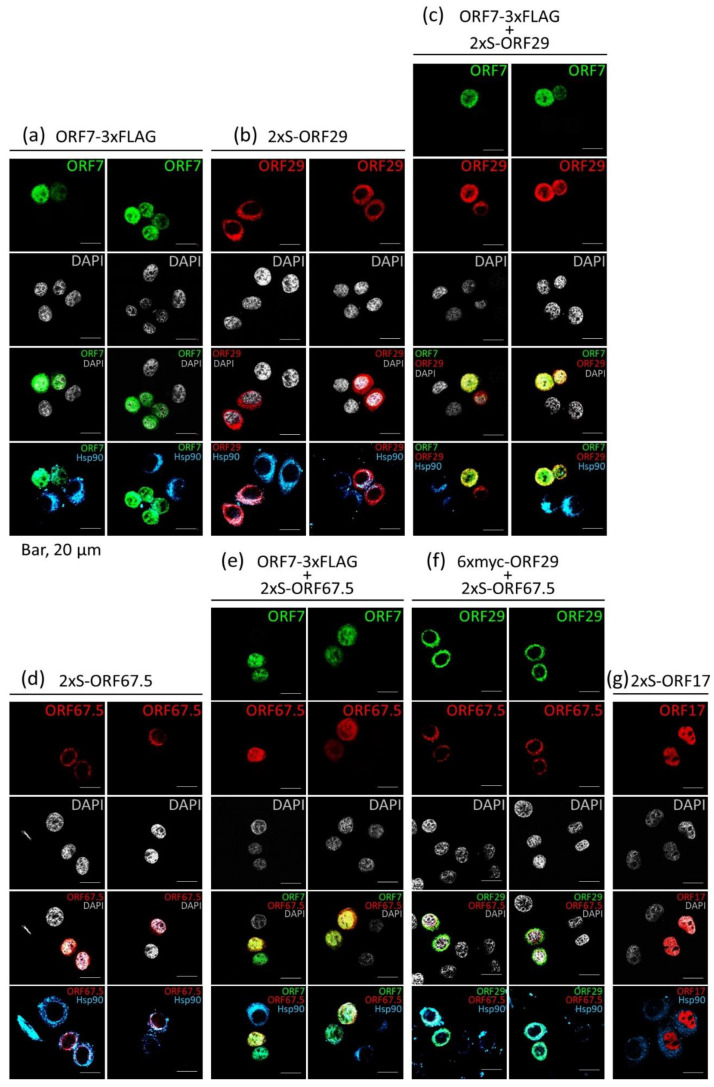
ORF7 induces nuclear relocalization of ORF29 and ORF67.5. HeLa cells were transfected with ORF7-3xFLAG (**a**), 2xS-ORF29 (**b**), 2xS-ORF67.5 (**d**), and 2xS-ORF17 (**g**) plasmids and stained with anti-HSP90beta (cyan) and either anti-FLAG (green) or anti-S tag (red) antibodies. (**a**–**g**) DNA was visualized with DAPI staining (white). Immunofluorescent images were obtained with an inverted confocal microscope. For colocalization with ORF7, ORF7-3xFLAG plasmids were cotransfected with 2xS-ORF29 (**c**) or 2xS-ORF67.5 (**e**) plasmids into HeLa cells. The cells were stained with anti-FLAG (green), anti-S tag (red), and anti-HSP90beta (cyan) antibodies. For colocalization of ORF29 and OR67.5 (**f**), 6xmyc-ORF29 was cotransfected with 2xS-ORF67.5, and HeLa cells were stained with anti-myc (green), anti-S tag (red), and anti-HSP90beta (cyan) antibodies.

It is speculated that during KSHV capsid maturation, synthesized concatemeric viral genomes are cleaved at the proper unit-length of the genome by the terminase complex, which comprises KSHV ORF7, ORF29, and ORF67.5. The cleaved genome has been reported to be inserted and packaged into the procapsid via the portal protein (ORF43) and capsid-associated tegument complex (ORF19, ORF32, and ORF64) [31], and the procapsid matures into nucleocapsid in the nuclei. KSHV ORF7, ORF29, and ORF67.5 are speculated to be components of KSHV terminase complex and homologous to HSV-1 UL28, UL15, and UL33, respectively. To obtain evidence for the participation of ORF7 in KSHV terminase complex, we investigated the colocalization of ORF7, ORF29, and ORF67.5 (Figure 4). We attempted to generate anti-ORF7 rabbit polyclonal antibody using GST-fused ORF7 as an antigen. However, we failed to prepare ORF7 protein, because GST-fused ORF7 protein was expressed in inclusion bodies in *E. coli* cells. Alternatively, we used 3xFLAG-tagged ORF7 expression plasmid.

First, we analyzed the localization in HeLa cells of exogenously transfected ORF7-3xFLAG (a), 2xS-ORF29 (b), 2xS-ORF67.5 (d), or 2xS-ORF17 alone (g), using an IFA. The localization of DNA (nucleus), HSP90b (cytoplasm), ORF7-3xFLAG, and 2xS-ORFs (ORF29, ORF67.5, and ORF17) is represented using white, cyan, green, and red, respectively. We observed that exogenous ORF7 expression led to a change in cell morphology, from a flat adhesive type to a sphere (Figure 4a). Although ORF7-expressing spherical cells (green) showed less cytosolic area compared with ORF7-nonexpressing flat adhesive cells, exogenous ORF7 localized to both cytosol and nuclei in HeLa cells. On the other hand, exogenously expressed ORF29 and ORF67.5 (red) localized to the cytosol (Figure 4b,d). KSHV ORF17 (homologous to HSV-1 UL26) is a fusion-precursor viral protease comprising viral protease (HSV-1 VP24) and assembly protein (HSV-1 VP21). UL26 (precursor viral protease) is known to be localized in the nuclei [32]; therefore, ORF17 staining (red) was presented as a positive control of nuclear localization (Figure 4g). Next, ORF7-3xFLAG plasmids were cotransfected with either 2xS-ORF29 or 2xS-ORF67.5 plasmids into HeLa cells, and the localization of each was analyzed. Interestingly, ORF29 and ORF67.5 (red) were predominantly relocalized into nuclei from the cytosol by the coexpression of ORF7 (green). In addition, ORF29 and ORF67.5 mainly colocalized with ORF7 in nuclei (stained yellow in Figure 4c,e). ORF7 (green) was also relocalized into nuclei by coexpression of either ORF29 or ORF67.5. We furthermore analyzed whether ORF29 and ORF67.5 were colocalized in the nuclei. HeLa cells were exogenously transfected with 6xmyc-ORF29 (green) and 2xS-ORF67.5 (red) and then analyzed. Both ORF29 and ORF67.5 localized in the cytosol. These findings indicate that ORF7 induces nuclear relocalization of ORF29 and ORF67.5 from the cytosol, and ORF7, ORF29, and ORF67.5 are colocalized into the nuclei by nuclear-localized ORF7. We speculate that ORF7 probably acts in concert with ORF29 and ORF67.5 as a terminase complex in the nuclei.

### 3.5. ORF7 Interacts with Both ORF29 and ORF67.5, Whereas ORF29 and ORF67.5 Fail to Interact with Each Other

**Figure 5:**
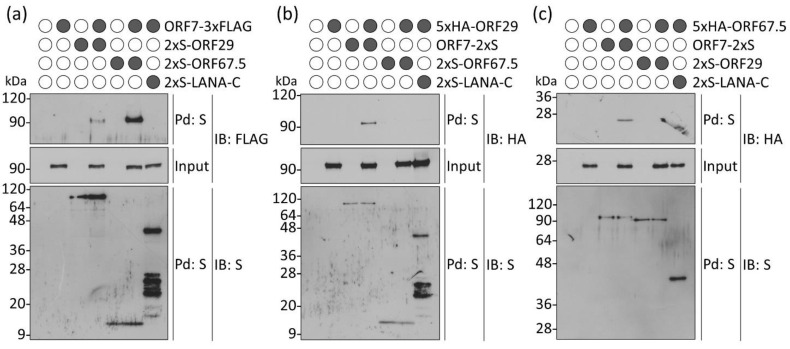
ORF7 functions as a hub molecule for interactions between ORF29 and ORF67.5. (**a**) ORF7 interacted with ORF29 and ORF67.5. HEK293T cells were cotransfected with plasmids expressing ORF7-3xFLAG and either 2xS-ORF29 or 2xS-ORF67.5. Cell lysates prepared at 18 h post-transfection were subjected to a pulldown assay (Pd) using S-protein beads. The precipitated 2xS-ORF29 or 2xS-ORF67.5 was analyzed by immunoblotting (IB) using anti-FLAG antibody to detect ORF7-3xFLAG. (**b**) ORF29 interacted with ORF7 but not ORF67.5. Cells were cotransfected with 5xHA-ORF29 and either ORF7-2xS or 2xS-ORF67.5 plasmids. The precipitated ORF7-2xS or 2xS-ORF67.5 was analyzed by IB using anti-HA antibody to detect 5xHA-ORF29. (**c**) ORF67.5 interacted with ORF7 but not ORF29. Cells were cotransfected with 5xHA-ORF67.5 and either ORF7-2xS or 2xS-ORF29 plasmids. The precipitated ORF7-2xS or 2xS-ORF29 was analyzed by IB using anti-HA antibody to detect 5xHA-ORF67.5. (**a**–**c**) “Input” means total cell lysates, and 2% cell lysates used for pulldown were applied for SDS-PAGE. 2xS-LANA-C plasmid (KSHV ORF73/LANA 954-1162 C-terminal end) was used for the negative control for ORF7 binding. Original images of blotting are shown in Supplementary Figures S3 to S5.

To gain further insights into the interactions among KSHV terminase components, we first analyzed the binding between ORF7 and other components (ORF29 and ORF67.5). HEK293T cells were cotransfected with ORF7-3xFLAG and either 2xS-ORF29 or 2xS-ORF67.5, and cells harvested at 18 h post-transfection were subjected to a pulldown assay. The data showed that exogenous ORF7 clearly interacted with exogenous ORF29 and ORF67.5 (Figure 5a). Next, we analyzed the binding between ORF29 and other components (ORF7 and ORF67.5). The data showed that ORF29 interacted with ORF7, whereas ORF29 failed to interact with ORF67.5 (Figure 5b). Finally, we analyzed the binding between ORF67.5 and other components (ORF7 and ORF29). As expected, the data showed that ORF67.5 interacted with ORF7 but not ORF29 (Figure 5c). ORF7 interacted with ORF29 and ORF67.5, whereas no interaction between ORF29 and ORF67.5 was detected. These findings indicate that ORF7 interacts with both ORF29 and ORF67.5, whereas ORF29 and ORF67.5 fail to interact with each other, suggesting that ORF7 functions as a hub molecule in the KSHV terminase complex for interactions between ORF29 and ORF67.5. These results, together with our colocalization data (Figure 4), provide further evidence for the participation of ORF7 with ORF29 and ORF67.5 in the KSHV terminase complex.

## 4. Discussion

We evaluated viral replication in ORF7-deficient KSHV-producing iSLK cells, which were stably integrated with a ΔORF7-KSHV BAC clone. ΔORF7-KSHV attenuated viral production compared with WT-KSHV and revertant KSHV, whereas there were no significant differences in replication of viral DNA among WT-KSHV, ΔORF7- KSHV, and revertant KSHV (Figure 2). The complementation assay showed that the recovery level of virus production in ORF7-complemented cells harboring ΔORF7-KSHV was nearly equal to that of cells harboring WT-KSHV (Figure 3). These data indicate that ORF7 plays an important role in virus production in the KSHV lytic cycle, which is in good agreement with findings in other herpesviruses. ORF7 of KSHV (of the gamma-herpesvirus subfamily) is homologous to UL28 of HSV-1 (alpha-herpesvirus subfamily) and UL56 of HCMV (beta-herpesvirus subfamily). UL28-KO HSV-1 and UL56-KO HCMV also showed decreased virus production but not viral replication [24,33,34]. Although Epstein–Barr virus (EBV) and KSHV belong to the same gamma-herpesvirus subfamily, EBV BALF3, which is homologous to KSHV ORF7, is essential for both virus production and replication of viral DNA [35]. It is interesting that EBV shows a different phenotype from that of KSHV, HCMV, and HSV-1; however, the reason for this difference is unknown. It is possible that EBV BALF3 may have additional (or different) functions in viral replication.

The anti-HCMV compound letermovir (AIC246, MK-8228), which exhibits highly anti-HCMV activity both in vitro and in vivo, is thought to inhibit the UL56 subunit of the terminase complex, resulting in a decrease in viral DNA cleavage and packaging into the capsid [36]. During nucleocapsid formation (capsid maturation), the cleavage of viral DNA genomes and packaging of the viral genome into the capsid cavity are virus-specific processes that are essential for virus production. Moreover, these processes are mediated by virus-specific enzymes. Thus, virus-specific enzymes, such as terminase, could be good targets for the development of antiviral drugs. As KSHV ORF7 is essential for virus production, the importance of KSHV ORF7 in lytic replication is in agreement with similar findings for homologous genes in other herpesviruses [24,33,34,35]. Therefore, it could be speculated that KSHV ORF7 represents a promising target molecule for any anti-KSHV drugs under development.

In the case of HSV-1, the terminase complex mediates the cleavage of replicated concatemeric viral genome into an appropriate unit-length genome and genome packaging into immature capsids. Each of the terminase components, UL28 (KSHV ORF7 homolog), UL15 (ORF29 homolog), and UL33 (ORF67.5 homolog), is essential for concatemeric viral genome cleavage and genome packaging into procapsids [18,22,37]. UL28, UL15, and UL33 form the terminase complex, in which UL28 binds to UL15 and UL33 [20,21,22]. UL28 was shown to localize in cytosol if just UL28 was expressed, whereas it was relocalized into nuclei from the cytosol when UL15 and UL28 were coexpressed [38]. UL28 and UL33 were reported to be localized in the cytosol [38,39], whereas UL15 was reported to be localized in the nuclei [38]. In addition, UL15 induced relocalization of UL28 and UL33 into the viral DNA replication compartments within nuclei [39]. As for the binding correlation of each component, UL15 interacted with UL28 but not UL33 [40], and UL33 interacted with UL28 [40]. As no interaction between UL15 and UL33 could be detected, UL28 is thought to function as a hub for interactions between UL15 and UL33. The colocalization behaviors of KSHV terminase components are similar to those of HSV-1 terminase components; however, the subcellular localizations of KSHV terminase components are likely to be different from those of HSV-1. Our data show that exogenous ORF7 localized in both cytosol and nuclei. Exogenous ORF29 and ORF67.5 localized to the cytosol. However, ORF29 and ORF67.5 predominantly relocalized to the nuclei during ORF7 coexpression. In contrast with the localization pattern, the interaction behaviors of KSHV terminase components are in good agreement with those of HSV-1 terminase components. ORF7 interacted with ORF29 and ORF67.5 in the pulldown assay. These data suggest that ORF7 may act in concert with ORF29 and ORF67.5 during KSHV capsid maturation in the nuclei. In contrast with HSV-1 terminases, the functions of ORF7, ORF29, and ORF67.5 remain unknown. Although this study showed that ORF7 plays a critical role in virus production and interacts with ORF29 and ORF67.5, further investigations are needed to fully understand the detailed functions of ORF7, ORF29, and ORF67.5.

In conclusion, ORF7-deficient KSHV exhibited attenuated viral production, but there was no effect on viral genome replication. ORF7 was colocalized with other terminases, ORF29 and ORF67.5, in the nuclei. Our findings shed light on the importance of ORF7 for viral production during the KSHV lytic cycle. To the best of our knowledge, this is the first report showing the importance of ORF7 in the lytic cycle, using ORF7-deficient KSHV. Our findings suggest that the development of inhibitory compounds that suppress ORF7 function or the association between ORF7 and other terminases may represent a new strategy for the treatment of KSHV-related diseases in the future.

## Figures and Tables

**Table 1:** Capsid and capsid-related molecules of HSV-1 and KSHV.

Common Name	HSV-1	KSHV
capsid maturation protease	UL26 (VP24, VP21)	ORF17
assembly protein	UL26.5	ORF17.5
major capsid protein	UL19	ORF25
triplex protein 1	UL38	ORF62
triplex protein 2	UL18	ORF26
small capsid protein	UL35	ORF65
DNA packaging terminase subunit 1	UL15	ORF29
DNA packaging terminase subunit 2	UL28	ORF7
DNA packaging protein	UL33	ORF67.5
portal protein	UL6	ORF43
portal capping protein	UL25	ORF19
DNA packaging tegument protein	UL17	ORF32
large tegument protein	UL36	ORF64

**Table 2:** Primers for BAC mutagenesis, construction of expression plasmids, real-time PCR, and RT real-time PCR.

Primer Name	Primer Sequences (5′ -> 3′)
**BAC Mutagenesis *^a^***	
S_dORF7_fs_EP	ctctctgacctggatttgtagttgtgtacccgtaacgatg*caaaggaactggcggcggtcTAGGGATAACAGGGTAATCGATTT
As_dORF7_fs_EP	agggctgacacatcggcatagaccgccgccagttcctttg*catcgttacgggtacacaacGCCAGTGTTACAACCAATTAACC
S_dORF7_st_EP	gtgtctgactcccaaccagggcaccagtctgcaggccatgTAGTTAGATAGTctcccagacacggcctgcagTAGGGATAACAGGGTAATCGATTT
As_dORF7_st_EP	atgcgggggtacatatgtgactgcaggccgtgtctgggagACTATCTAACTAcatggcctgcagactggtgcGCCAGTGTTACAACCAATTAACC
S_dORF7_fs-rev_EP	ctctctgacctggatttgtagttgtgtacccgtaacgatggcaaaggaactggcggcggtcTAGGGATAACAGGGTAATCGATTT
As_dORF7_fs-rev_EP	agggctgacacatcggcatagaccgccgccagttcctttgccatcgttacgggtacacaacGCCAGTGTTACAACCAATTAACC
S_dORF7_st-rev_EP	gtgtctgactcccaaccagggcaccagtctgcaggccatgctcccagacacggcctgcagTAGGGATAACAGGGTAATCGATTT
As_dORF7_st-rev_EP	atgcgggggtacatatgtgactgcaggccgtgtctgggagcatggcctgcagactggtgcGCCAGTGTTACAACCAATTAACC
**Expression Plasmid *^b^***	
S_EcoRI_ORF7	catGAATTCatggcaaaggaactggcggc
As_XbaI_ORF7	gtcTCTAGAgacctgggagtcattgtggttgc
S_EcoRI_ORF17	catGAATTCatggcacagggcctgtacgtc
As_SalI_ORF17	tagGTCGACctactgcttgttcaggagctc
S_EcoRI_ORF29	catGAATTCatgctgctcagccgtcacag
As_SalI_ORF29	taaGTCGACttattgtggggatatgggcttgtac
S_EcoRI_ORF67.5	catGAATTCatggagtacgcgtctgaccag
As_SalI_ORF67.5	tgaGTCGACtcagggccgtgc
**Real-Time PCR**	
KSHV ORF11-F	TTGACAACACGCACCGCAAG
KSHV ORF11-R	AAAAATCAGCACGCTCGAGGAG
**RT Real-Time PCR**	
Fw-GAPDH	CATCAAGAAGGTGGTGAAGCAG
Rv-GAPDH	TGTCGCTGTTGAAGTCAGAGG
Fw-ORF16	AGATTTCACAGCACCACCGGTA
Rv-ORF16	CCCCAGTTCATGTTTCCATCGC
Fw-ORF47	CGATCCGAATCACTGCAACG
Rv-ORF47	CTGCTGCTTTTAGCCCGAG
Fw-K8.1	TCCCACGTATCGTTCGCATTTGG
Rv-K8.1	GCGTCTCTTCCTCTAGTCGTTG

*^a^* Asterisk (*) indicates one nucleoside deletion site. Lowercase, underlined uppercase indicates mutagenesis site, and uppercase indicates pEP-KanS sequence. *^b^* Uppercase indicates restriction enzyme site.

## Data Availability

DNA sequence data are available from the corresponding author upon reasonable request. Other data are presented in the manuscript.

## Supplementary Materials

The following are available online at https://www.mdpi.com/article/10.3390/microorganisms9061169/s1, Supplementary Figure S1 (original data of Figure 1c), Figure S2a (effect of exogenous ORF7 expression on lytic replication in wild-type KSHV-containing iSLK cells), Figure S2b (original data of Figure 3), Figure S3 (original data of Figure 5a), Figure S4 (original data of Figure 5b) and Figure S5 (original data of Figure 5c).
