# Supplementary material for: Kaposi’s Sarcoma-Associated Herpesvirus ORF7 Is Essential for Virus Production
Source: Microorganisms. 2021 May 28;9(6):1169. doi: 10.3390/microorganisms9061169 (PMC8228664; doi:10.3390/microorganisms9061169)
Supplement: Supplementary file 1 [file microorganisms-09-01169-s001.zip › microorganisms-1217141-supplementary.pdf]

Supplementary Figure S1
(original data of Figure 1c)

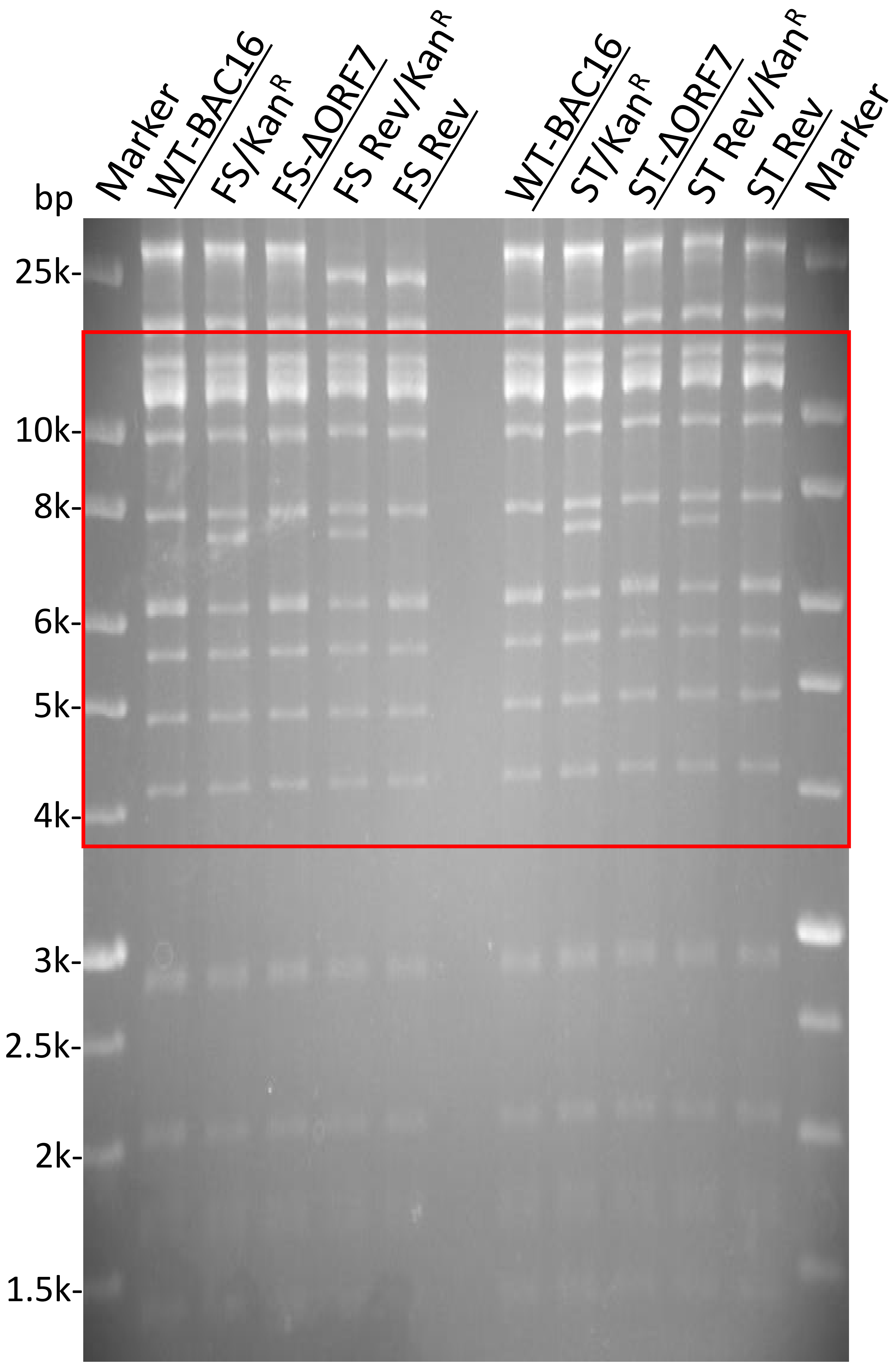

Supplementary Figure S2

(a) ORF7 expression does not influence the lytic replication of KSHV.

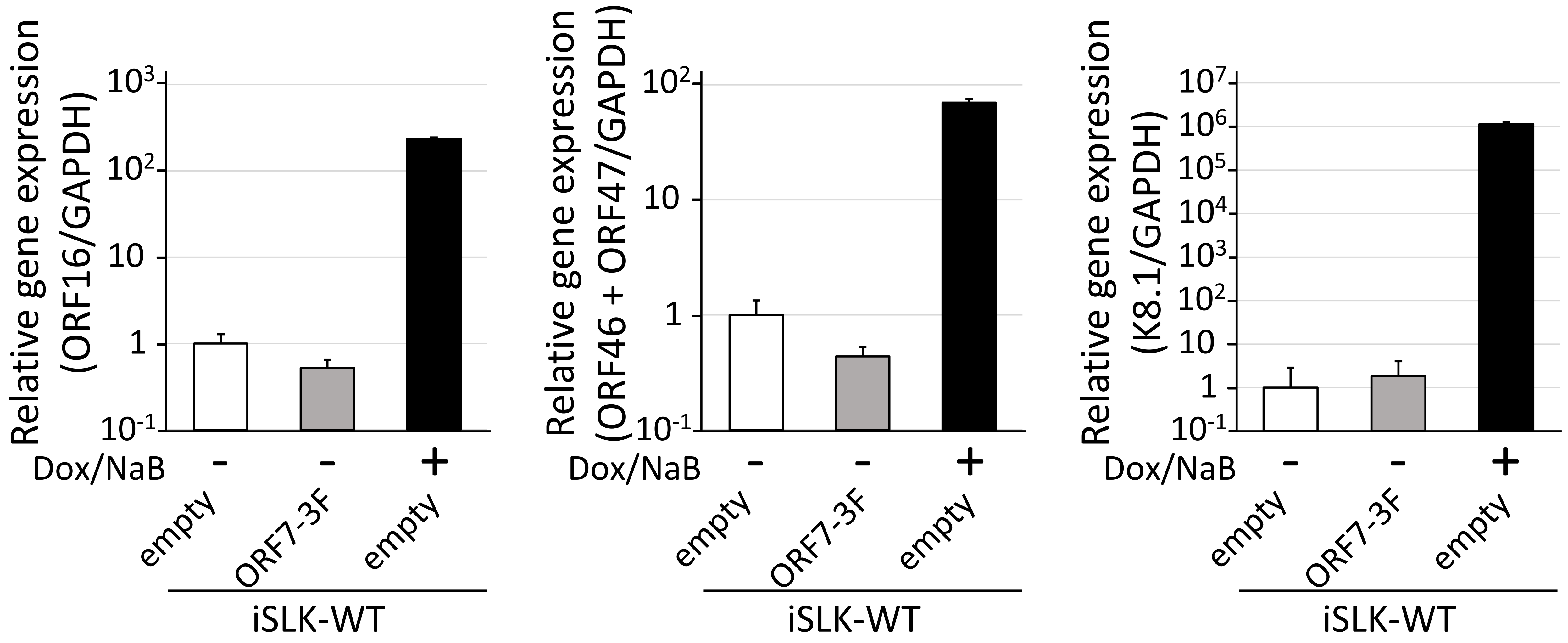

iSLK cells harboring WT-KSHV-BAC16 (iSLK-WT) were transfected with OFR7-3xFLAG (or empty) plasmid and cultured for 72 h. As the positive control, iSLK-WT cells were cultured for 72 h with medium containing 8 µg/mL Dox and 1.5 mM NaB to induce lytic replication. At 72 h post-transfection (or treatment), total RNA were purified from harvested cells were subjected to RT real-time PCR. The mRNA expression levels of KSHV lytic genes, ORF16 (immediate early), ORF46 and ORF47 (early), and K8.1 (late) were normalized by GAPDH mRNA. The values obtained from Dox- and NaB-untreated and empty plasmid-transfected iSLK-WT cells were defined as 1.0.

(b) original data of Fig.3

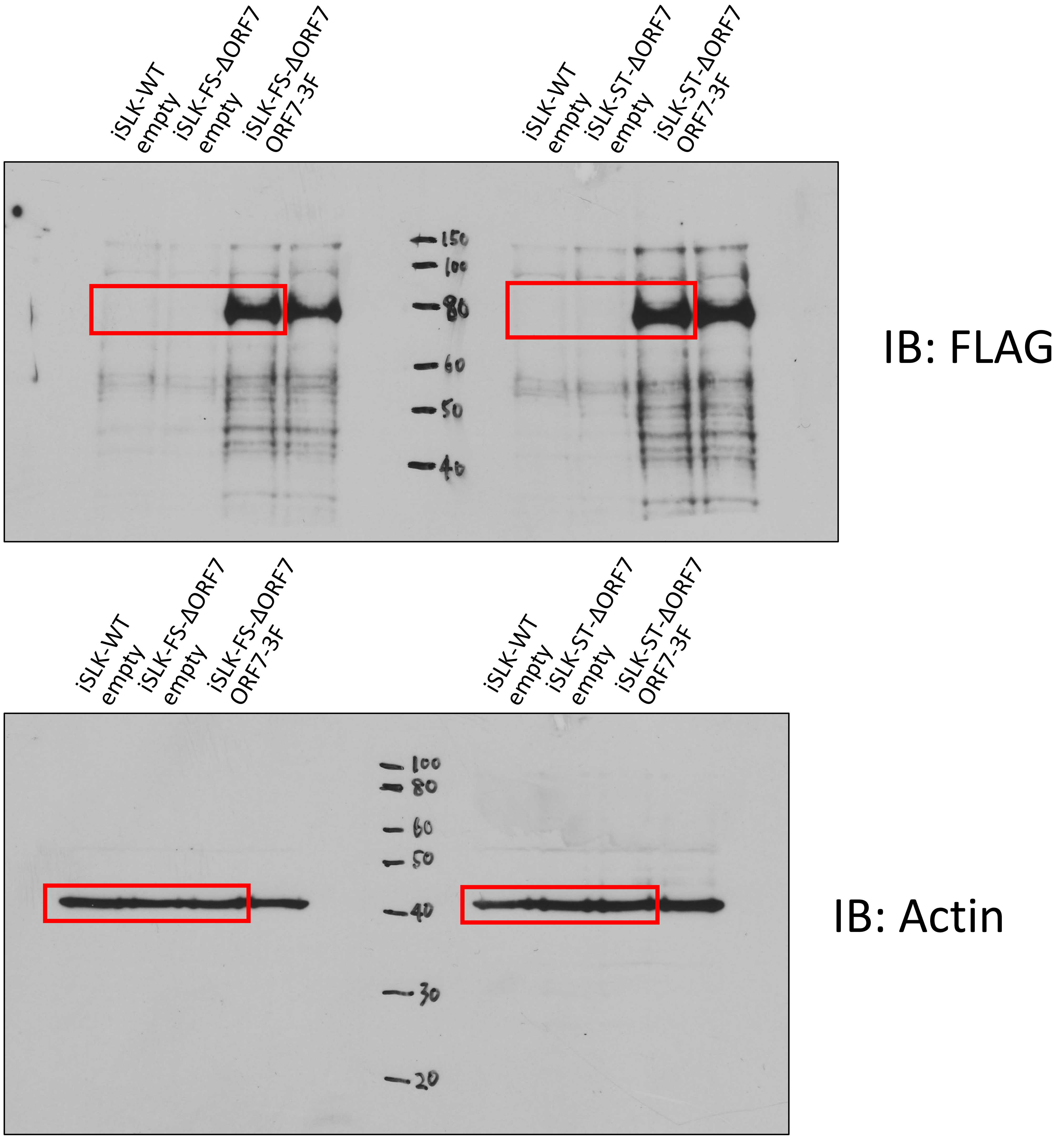

Supplementary Figure S3
(original data of Figure 5a)

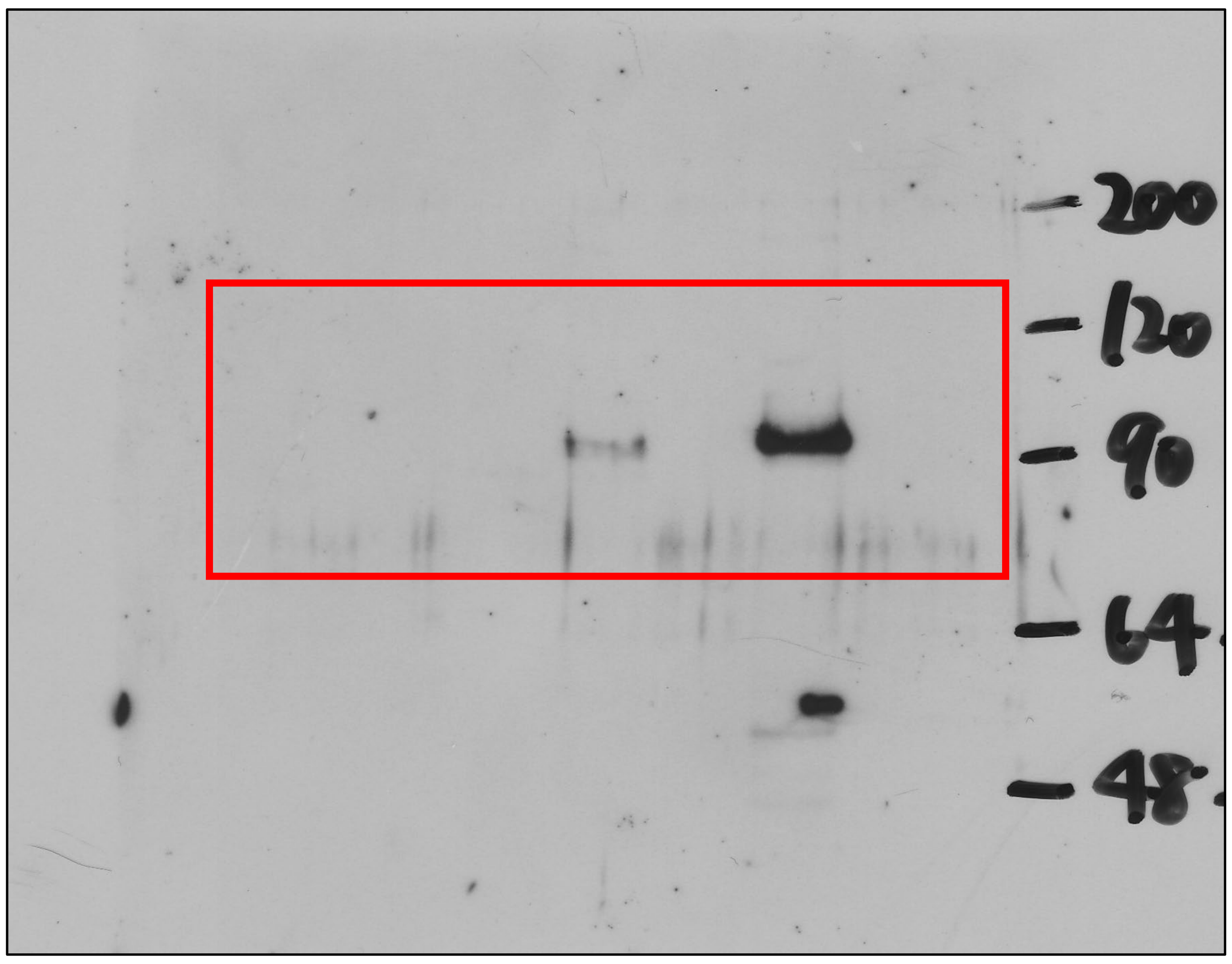

Pd: S
IB: FLAG

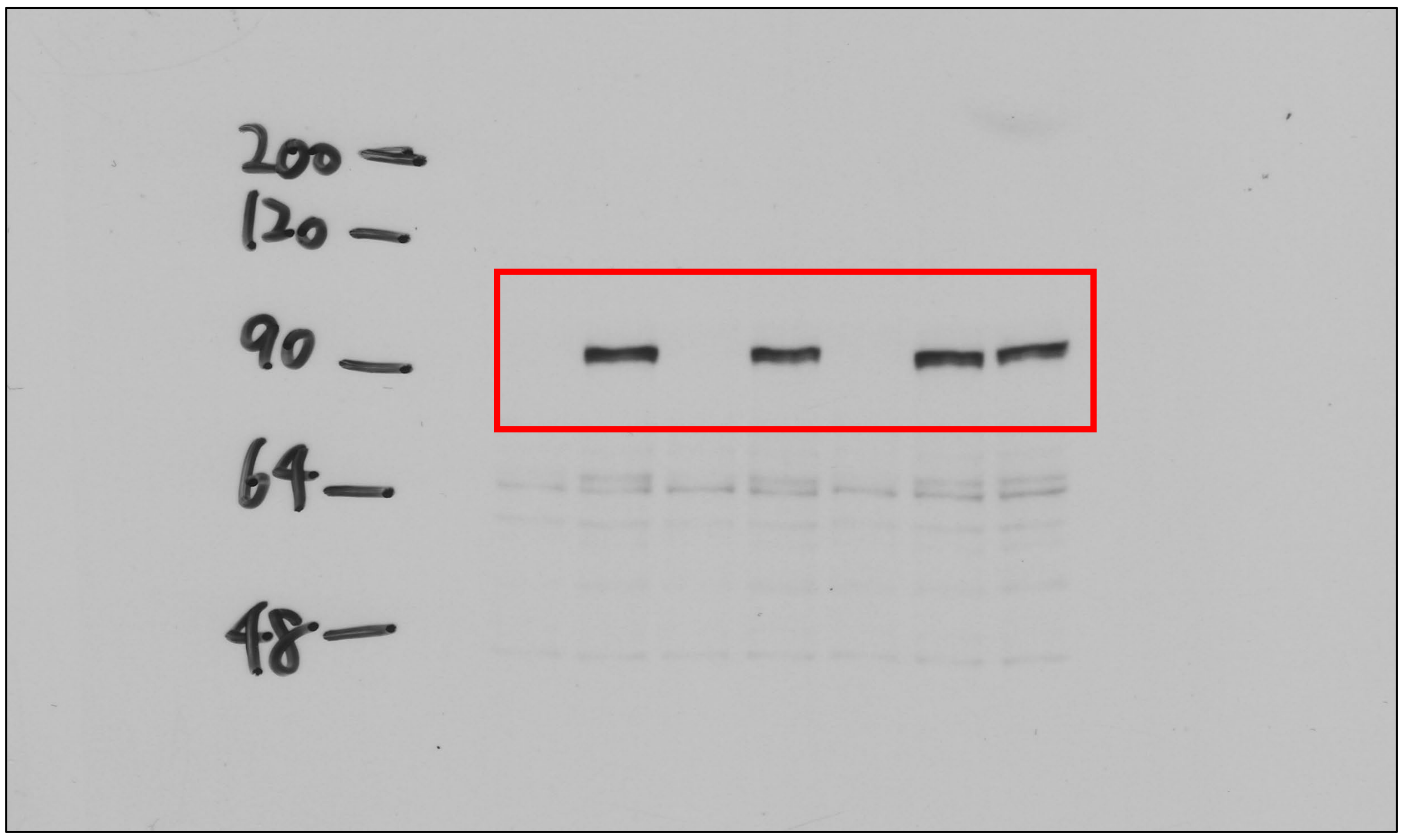

Input
IB: FLAG

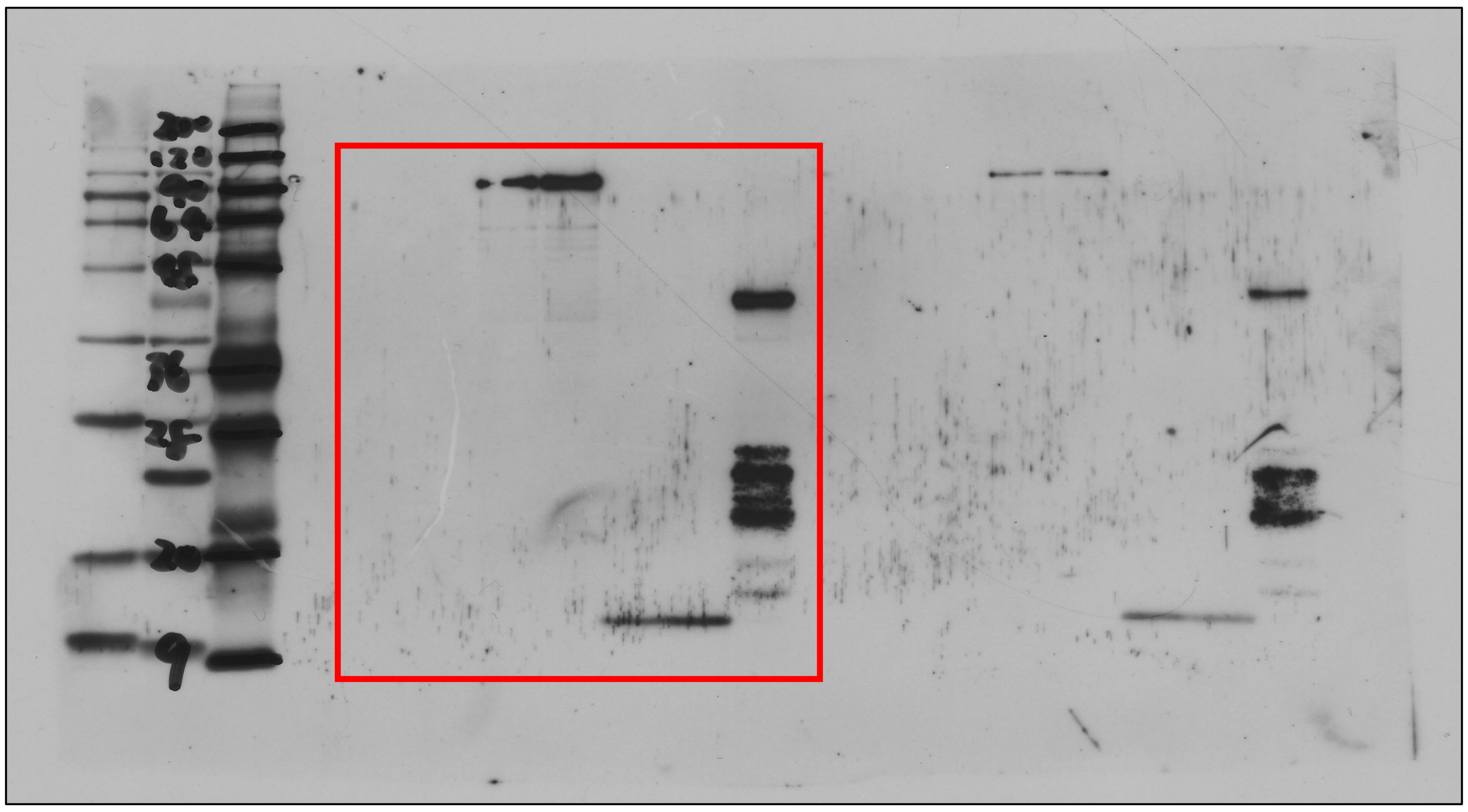

Pd: S
IB: S

Supplementary Figure S4
(original data of Figure 5b)

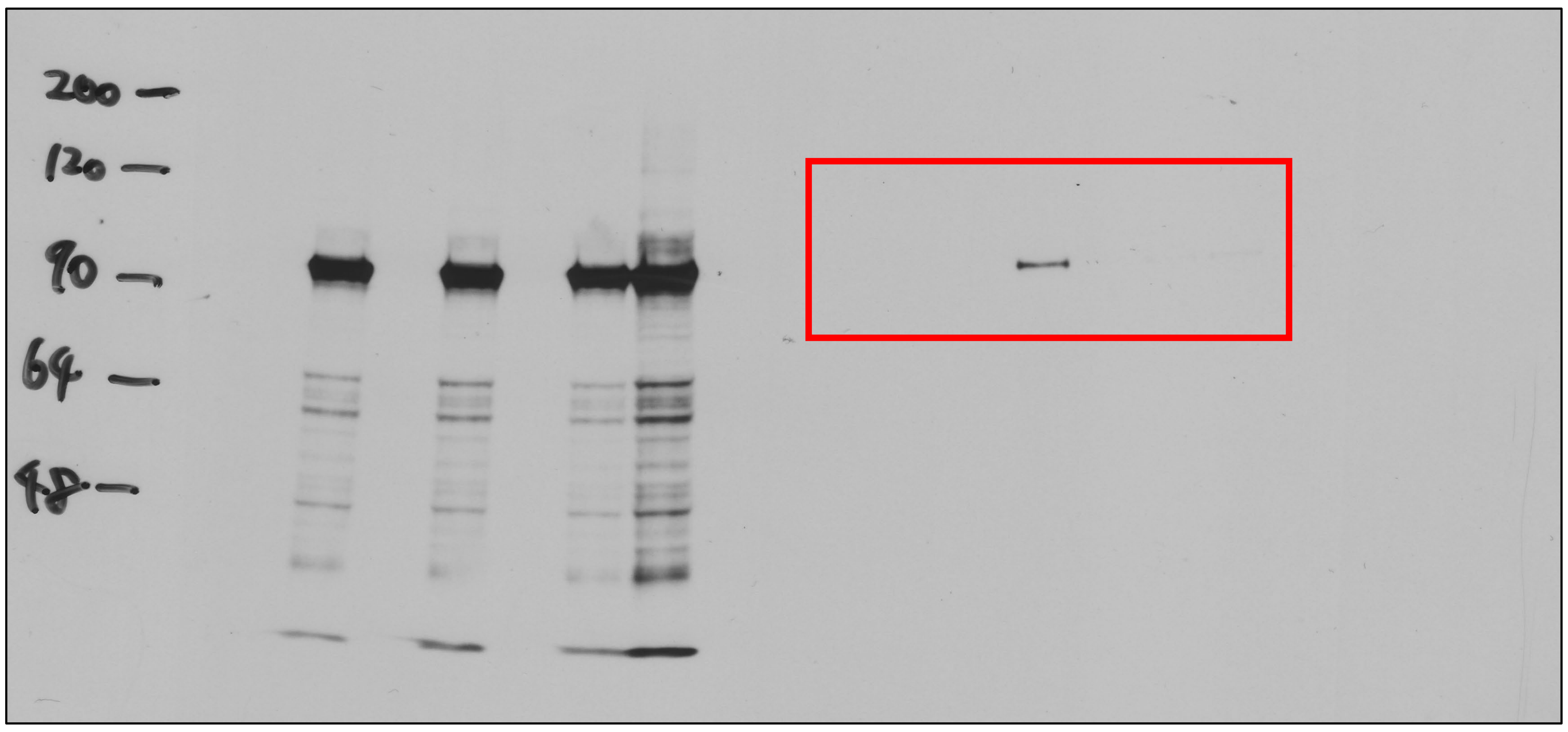

Pd: S
IB: HA

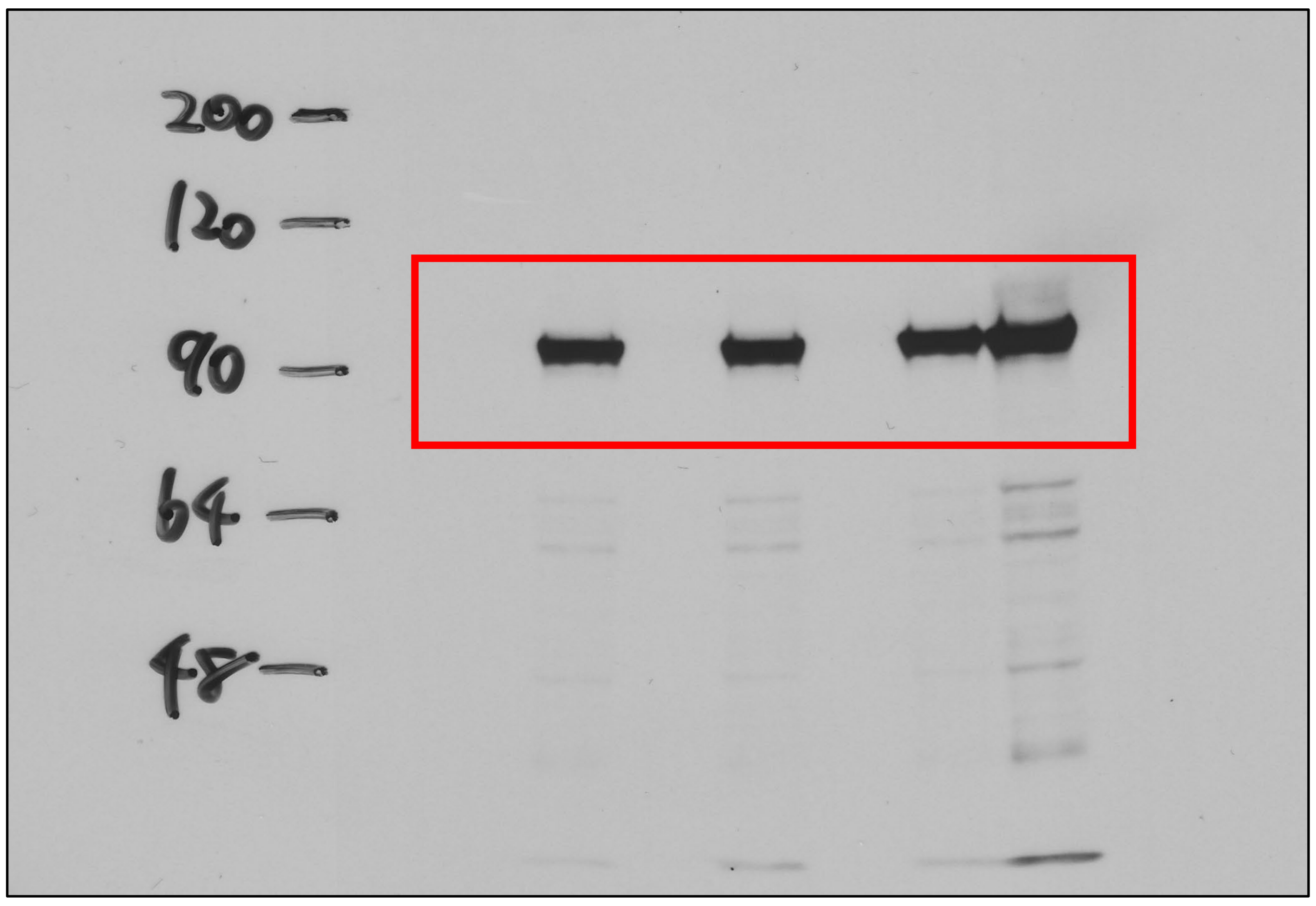

Input
IB: HA

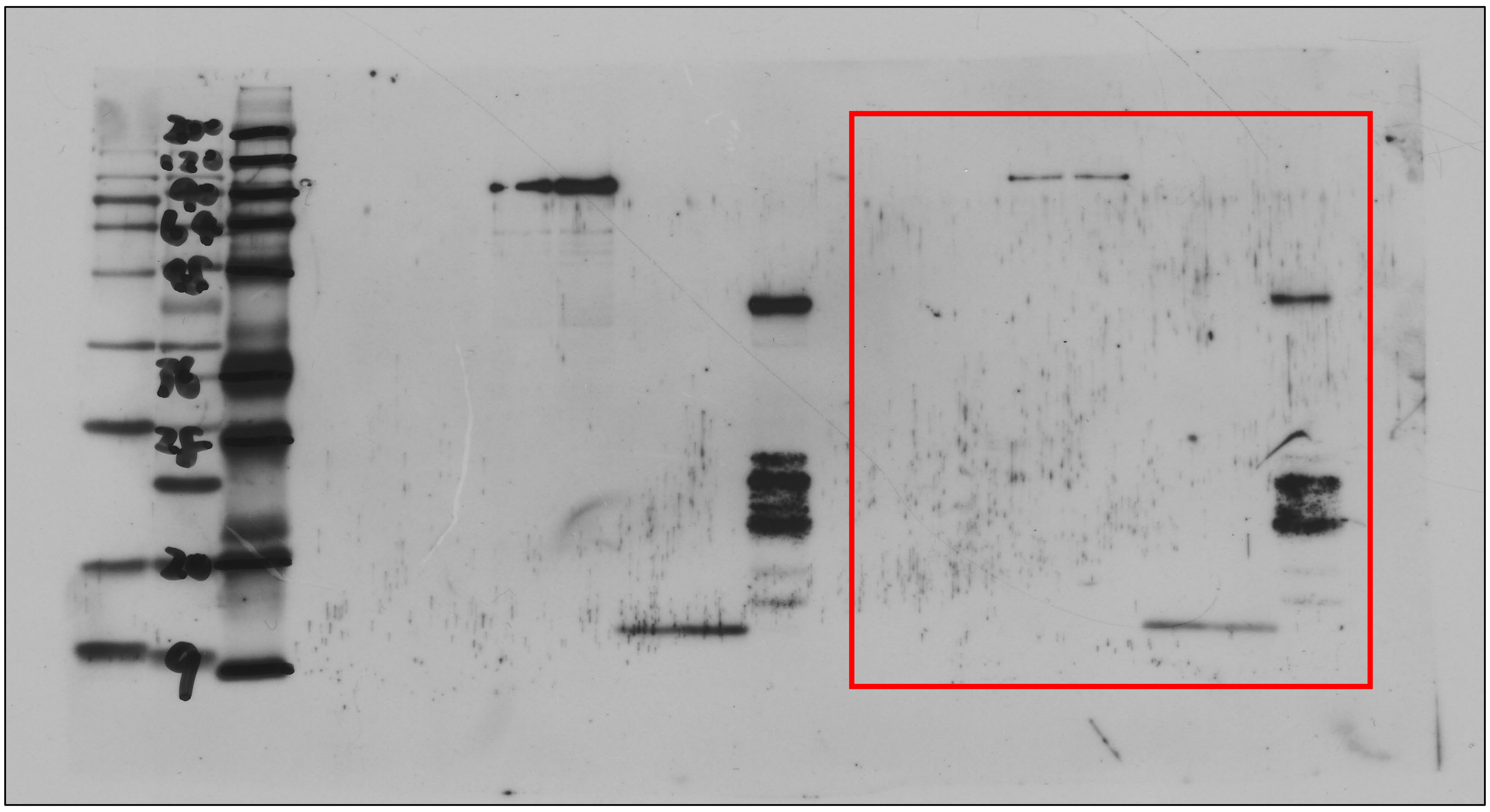

Pd: S
IB: S

Supplementary Figure S5
(original data of Figure 5c)

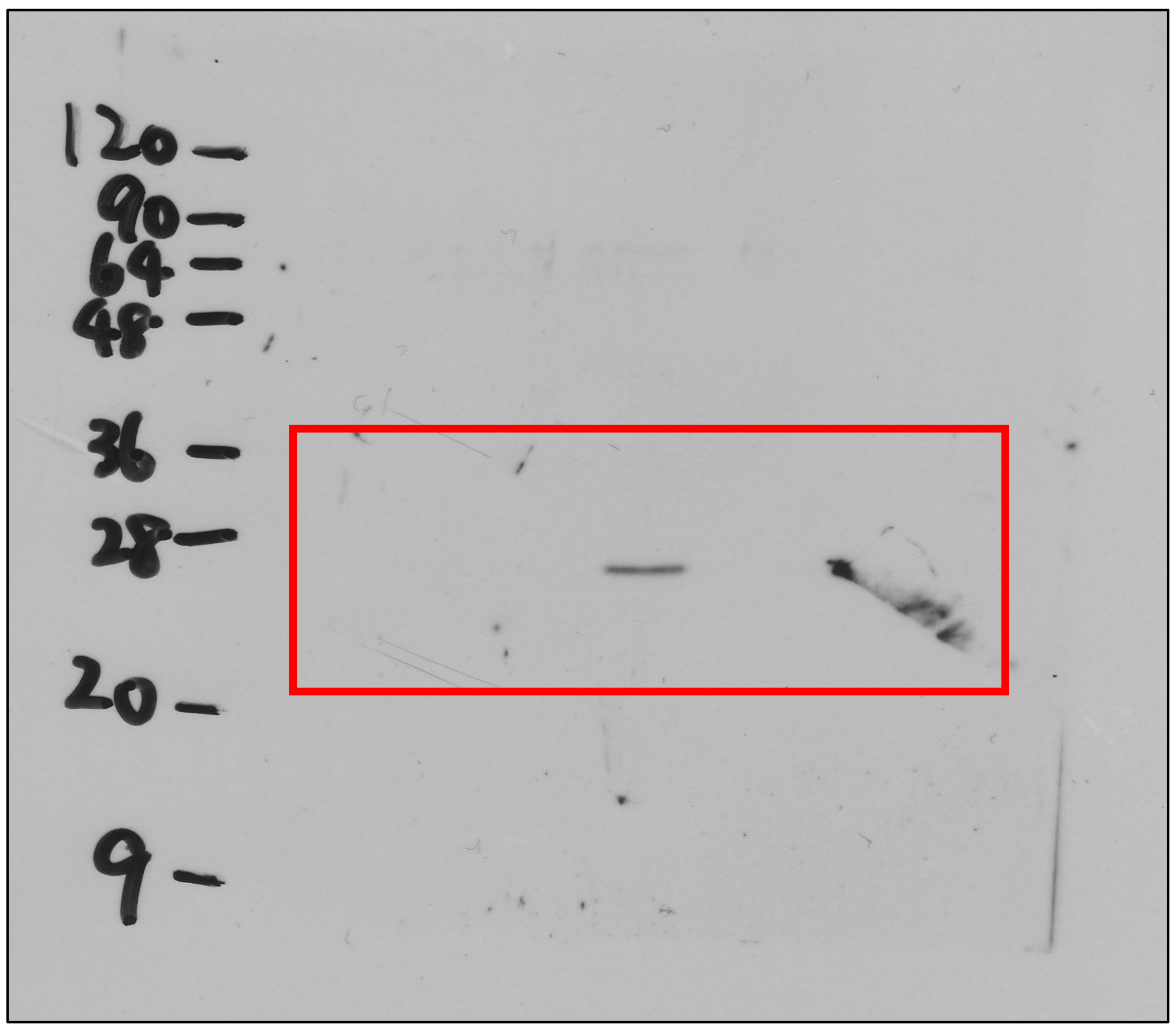

Pd: S
IB: HA

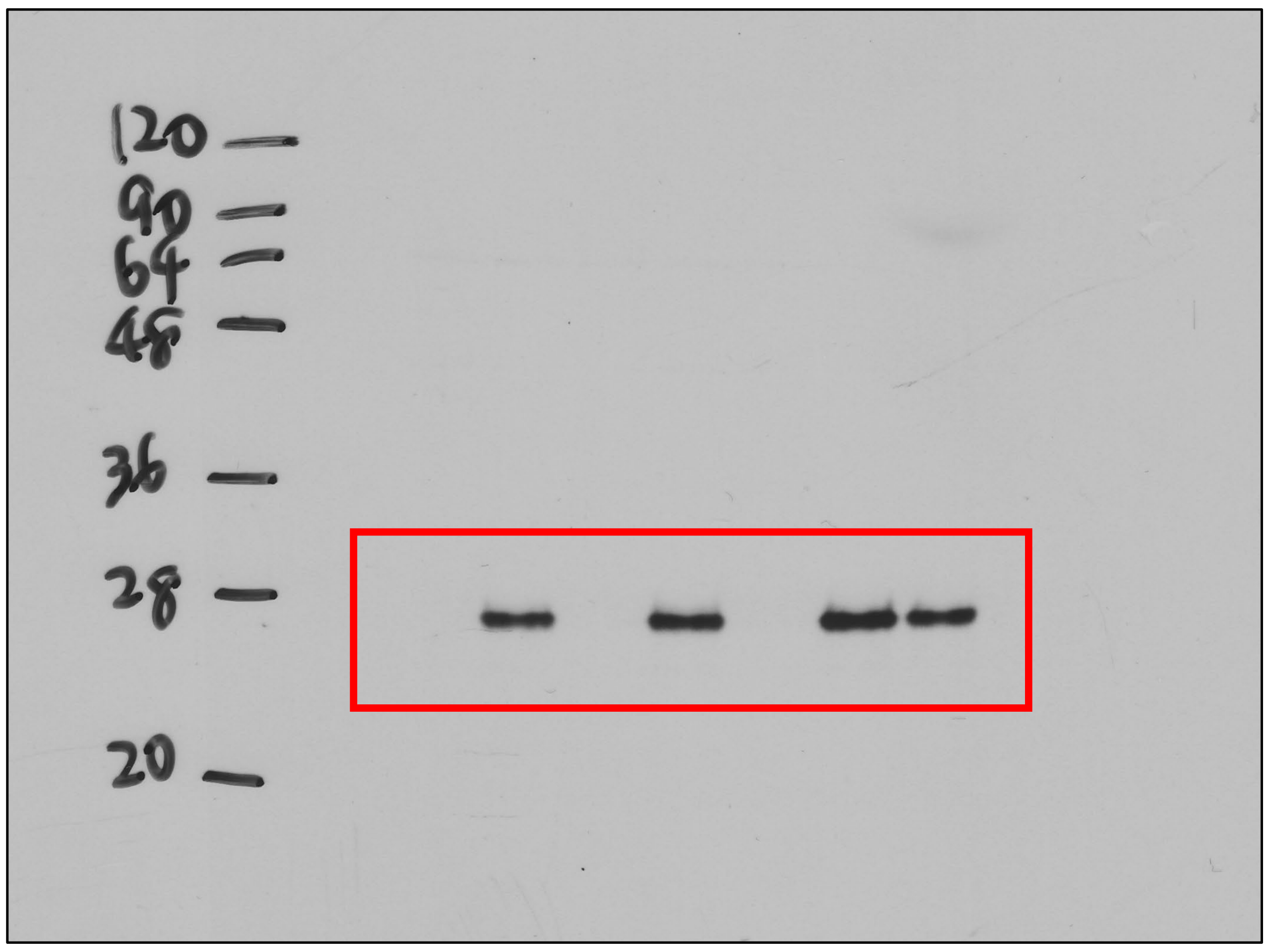

Input
IB: HA

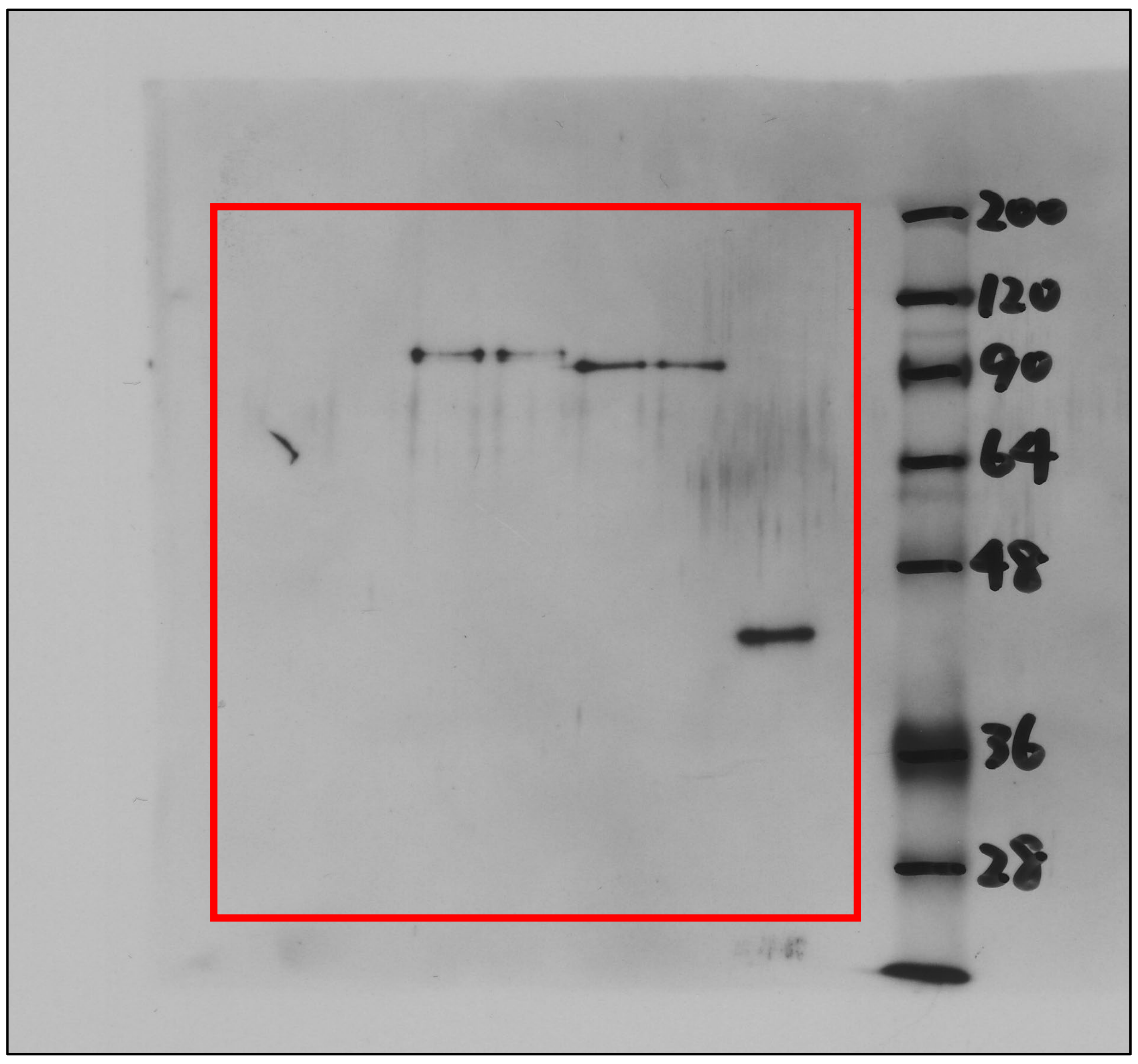

Pd: S
IB: S
